# The role of health extension workers in improving utilization of maternal health services in rural areas in Ethiopia: a cross sectional study

**DOI:** 10.1186/1472-6963-12-352

**Published:** 2012-10-08

**Authors:** Araya Medhanyie, Mark Spigt, Yohannes Kifle, Nikki Schaay, David Sanders, Roman Blanco, Dinant GeertJan, Yemane Berhane

**Affiliations:** 1 Department of Public Health, Mekelle University, Mekelle, Ethiopia; 2 Tigray Regional Health Bureau, Mekelle, Ethiopia; 3 Addis Continental Institute of Public Health, Addis Ababa, Ethiopia; 4 School of Public Health, University of Western Cape, Cape Town, South Africa; 5 Department of Medicine, University of Alcala de Henares, Madrid, Spain; 6 CAPHRI, School for Public Health and Primary Care, Maastricht University, Maastricht, Netherlands; 7 Department of General Practice, Tromso University, Tromso, Norway

**Keywords:** Health extension workers, Community health workers, Utilization, Maternal health services, Income generating activities

## Abstract

**Background:**

Community health workers are widely used to provide care for a broad range of health issues. Since 2003 the government of Ethiopia has been deploying specially trained new cadres of community based health workers named health extension workers (HEWs). This initiative has been called the health extension program. Very few studies have investigated the role of these community health workers in improving utilization of maternal health services.

**Methods:**

A cross sectional survey of 725 randomly selected women with under-five children from three districts in Northern Ethiopia. We investigated women’s utilization of family planning, antenatal care, birth assistance, postnatal care, HIV testing and use of iodized salt and compared our results to findings of a previous national survey from 2005. In addition, we investigated the association between several variables and utilization of maternal health services using logistic regression analysis.

**Results:**

HEWs have contributed substantially to the improvement in women’s utilization of family planning, antenatal care and HIV testing. However, their contribution to the improvement in health facility delivery, postnatal check up and use of iodized salt seems insignificant. Women who were literate (OR, 1.85), listened to the radio (OR, 1.45), had income generating activities (OR, 1.43) and had been working towards graduation or graduated as model family (OR, 2.13) were more likely to demonstrate good utilization of maternal health services. A model family is by definition a family which has fulfilled all the packages of the HEP.

**Conclusions:**

The HEWs seem to have substantial contribution in several aspects of utilization of maternal health services but their insignificant contribution in improving health facility delivery and skilled birth attendance remains an important problem. More effort is needed to improve the effectiveness of HEWs in these regards. For example, strengthening HEWs’ support for pregnant women for birth planning and preparedness and referral from HEWs to midwives at health centers should be strengthened. In addition, women’s participation in income generating activities, access to radio and education could be targets for future interventions.

## Background

In response to inadequate numbers of health personnel, many countries have focused on increasing production and distribution of personnel. This occurred in 1980s particularly in the community health worker cadre although in the 1990s many such programmes faltered [1]. Community health workers (CHWs) are widely used to provide care for a broad range of health issues. However, there is insufficient evidence about the effectiveness of their work in implementing comprehensive primary health care [2]. This lack of knowledge makes it difficult for policy makers to decide how CHWs can best improve the effectiveness of primary health care.

Like in many resource constrained countries, Ethiopia has been training and deploying different categories of volunteer community health workers (VCHWs) in the past decades. These CHWs include trained traditional birth attendants (TTBAs), community based reproductive health agents (CBRHAs) and community health agents (CHAs). However, to accelerate the expansion of primary health care coverage and to ensure equitable access to health services, the government of Ethiopia started deploying specially trained new cadres of community based health workers named Health Extension Workers (HEWs). This initiative has been called the Health Extension Program (HEP). The HEP has been introduced in recognition of the failure of essential services to reach the people at the grassroots level in particular to underserved rural population. It was designed based on the concept and principles of comprehensive primary health care [3-6].

The health system of Ethiopia is a four level health system, characterized by a primary health care unit (PHCU), and then the district hospital, zonal hospital and specialized hospital. A PHCU has been planned to serve 25,000 people, while a district and a zonal hospital are each expected to serve 250,000 and 1,000,000 people, respectively. Specialized hospitals are planned to serve a catchment area with 5 million people [7].

The lowest level in the Ethiopian health system is a PHCU, comprising one health center and five satellite health posts. Health centers are staffed with a health professional team including midlevel health professionals for instance health officers, nurses, midwives, sanitarians and laboratory technicians. A health center provides comprehensive primary health care which includes promotive, prevention, curative and rehabilitative services. One health center supervises and receives referrals from five satellite health posts. A health post is the operational center for two HEWs. On average, a health post serves a kebele which comprises approximately 1000 households or 5000 people.A kebele is the smallest administrative unit in Ethiopia, but it can include several small villages. By the year 2010, a total of about 34,000 HEWs were trained and deployed throughout the country. In addition about 15,000 health posts were constructed in the country. HEWs are required to spend 75% of their time conducting outreach activities by going from house to house in their respective kebele, while the rest of their time they are supposed to be at the health post. All HEWs have completed high school and received additional training for 1 year at an undergraduate level. The HEWs are also different from the VCHWs in that they are employed in the government health system structure and get monthly salary. They receive more advanced and comprehensive training when compared to VCHWs [3-6].

With the aim of reducing maternal mortality, HEWs are trained on how to provide care to pregnant mothers through pregnancy, birth and postnatal period. HEWs inform pregnant mothers on safe motherhood when they provide antenatal care (ANC), birth and post natal care (PNC). HEWs also provide family planning services and are trained on how to educate women on the use of iodized salt and HIV testing.

Since the implementation of the HEP, few studies have published findings on the effectiveness of HEWs [8]. These studies have shown their effectiveness in improving utilization of family planning and immunization services. However, none of them investigated the HEWs role in improving utilization of comprehensive maternal health services.

This study focuses on the extent to which these specially trained community health workers have contributed to the improvement of utilization of maternal health services by rural women in Ethiopia. We compare current utilization of maternal health services with the Ethiopian Demographic Health Survey 2005(EDHS 2005) and investigate which variables may be related to good utilization of maternal health services.

## Methods

### Study design

A cross-sectional survey was undertaken in August-November 2009 to assess utilization to maternal health services by women in rural villages in Ethiopia.

### Setting

The study was conducted in Tigray region, Ethiopia. Tigray is one of the nine regions and the northernmost regional state of Ethiopia. The 2007 Ethiopian census showed the population of the region to be 4.3 million of which 80% lived in rural areas and 51% were female [9]. The poor health status of Tigray region is comparable to the rest of the country, showing high infant mortality rate (67/1000), low institutional delivery (8.6%), high HIV prevalence (2.7%), and low family planning utilization (16.5%) [10]. This study was done in three rural districts. The districts studied were Alaje from the Southern Zone, Saesi Tsadamba from the Eastern Zone and Degua Tembien from the South Eastern Zone of Tigray. These districts were selected purposefully in consultation with Tigray regional health bureau. We considered accessibility of the districts to carry out the research in terms of transport. Out of the total 72 kebeles in these districts, 13 of them had health centers. Twelve of them did not have any health facility. The rest 47 rural kebeles were with only health post. From each selected district, three rural kebeles were selected. Rural kebeles with no functional health posts were excluded from the selection. Rural kebeles who have health facilities other than health posts were not also included in the study. All the selected kebeles were with functional health posts and HEWs.

### Sample size

We employed the Statcalc sample size calculation for cross-sectional study module of EPI-info version 2002 to determine sample size for our study. A total sample size of 726 households was determined by considering 95% confidence interval, 80% power of study; 1:1 comparison among districts, a contraceptive prevalence rate of 16.2% for Tigray region taken from EDHS 2005, and we assumed that the proportion of contraceptive users would be two times when we did our data collection in 2009.

### Study population

Women with under-five children from the nine selected kebeles who were willing and healthy enough to be interviewed were identified to participate in the survey. To select the study participants a sampling frame of households with women who had under-five children was developed from the log book of the HEWs. These log books of HEWs have a list of households in their kebeles. Using systematic random sampling, we selected an average of 80 women with under-five children from each kebele. There was no refusal to participate. When the woman selected for an interview was not available, a neighbouring woman was interviewed. A total of 726 women were interviewed and data from 725 women were included in the analysis; 1 questionnaire was useless because of its incompleteness.

### Data collection

We collected data on women’s utilization of family planning, antenatal care, delivery care, postnatal care, HIV testing and use of iodized salt. Data on women’s utilization of maternal health services by type of health workers were collected. The questionnaire was initially developed in English and then translated to the local language, *‘Tigrigna’*. The questionnaire was pre-tested among 20 mothers to assure clarity of concepts for respondents.

The data were collected by six data collectors who had completed high school and who had experience in doing questionnaire interviews. Additional training was given for the data collectors to help them understand the nature of the study and the questions. Completed questionnaires were checked for completeness and consistency at the time of interview by supervisors. To ensure rigor in the study, supervisors re-checked the responses for a randomly selected 5% of the questionnaires by going back to the woman’s house. Re-checking showed no major problems in data collection.

### Outcome variables and definitions

Utilization of maternal health services was collected using the following variables: 1. *Family planning*: whether the woman has been using contraceptives during the interview period (current utilization) or whether the woman has ever used contraceptives in her lifetime (ever utilization). 2. *Antenatal care (ANC)*: whether the woman attended a health facility for antenatal care (ANC) at least once in her last successful pregnancy. 3. *Health facility delivery*: whether the woman gave birth at a health facility for her youngest child. 4. *Postnatal care (PNC)*: whether a health professional or community health worker visited the woman at her home within 24 hours of the birth of her youngest child. 5. *HIV testing*: whether the woman had ever had an HIV test by the time of interview. 6. U*se of iodized salt*: whether iodized cooking salt (with 15 parts per million based on salt testing kits) was found in the woman’s house. This was considered as one of the maternal health services because educating women on utilization of iodized salt and distributing subsidized iodized salt are among the tasks of HEWs.

In addition we measured several other variables such as age, educational status, marital status, religion, year of enrolment into the HEP, household status in relation to working towards graduation or graduated as model family and participation in income generating activities (IGAs). A model family is by definition a family which has fulfilled all the packages of the HEP. Prior to the data collection we checked log books of HEWs on this information. We found the log books had incomplete and inconsistent information on whether a family completed all the packages of the program or not. Hence for this study we took the woman’s word whether she said her household had been working towards graduation or graduated as model family or not. IGAs are government or community-initiated activities for local people to earn some money. These IGAs include irrigation schemes, micro-finance credit, safety net, cattle rearing, poultry production and bee keeping.

### Data management and analysis

Frequencies of utilization of specific maternal health services were calculated. To estimate changes in the utilization of maternal health services over the years, we compared our findings with findings of the EDHS 2005. The EDHS 2005 was a nationally representative survey of 14,070 women aged 15–49. The data collection of this survey was conducted from April 27-August 30, 2005.

To investigate which factors were associated with good utilization of maternal health service, we used logistic regression. To calculate Adjusted Odds Ratios (AOR) we included all independent variables in one model. The dependent variable was computed by combining the six outcome variables. Using the mean (3.01) as a cutoff point, we categorized utilization of maternal health services into two categories. Women who had utilized 4 and more maternal health services were defined as having good utilization of maternal health services, while those who had utilized less than 4 were considered as having poor utilization of maternal health services. Women’s response for questions on religion, marital status and occupation were virtually the same. Therefore, these variables were not used in the analysis.

### Ethical considerations

The study was approved by the ethics committee at the College of Health Sciences of Mekelle University, Ethiopia which offered a letter with reference number CHS/236/A-16/09 dated on 05/March/09. Study participants were informed about the purpose of the study, anticipated benefits, how they were chosen to participate, data collection procedures and their full right to refuse, withdraw from part or all of the study. The participant’s name was kept confidential. Verbal informed consent was obtained from each study participant. Verbal consent instead of written consent was chosen as most of the questions in the survey were not sensitive and a great number of rural women in Ethiopia are unable to read and write.

## Results

### Respondents' characteristics

The mean age of the 725 study participants was 31.4 years (Table 1) and almost all (99.9%) were orthodox Christians. The mean number of children per woman was 4.15. The majority of participants (86%) participated in at least one income generating activity. Micro-finance credit and the safety net programs were the most reported IGAs.

**Table 1:** Respondents’ characteristics, September 2009 (N=725)

**Characteristics of respondents**	**Frequency (no./%)**
**Number of participants per district**	
District 1 : Degua Tembien	240(33.1)
District 2: Saesi Tsadamba	244(33.7)
District 3: Alaje	241(33.2)
**Age of respondents**	
24 and less	106(14.6%)
25 and above	619(85.4%)
**Educational level**	
Illiterate (unable to read and write)	576(79.4)
Illiterate (able to read and write)	149(20.6)
**Marital status**	
Yes, currently married	644(88.8%)
Not in a union	81(11.2%)
**Do you listen to radio**	
No	439(60.6)
Yes	286(39.4)
**Participation in income generating activities (IGAs)**	
Poor Participation: in 2 and less IGAs	456(62.9)
Good participation: in 3 and more IGAs	269(37.1)
**Year of enrolment in HEP**	
Do not know	159(21.9)
2004-2006	238(32.8)
2007-2009	328(45.2)
**Household status towards graduation as model family**	
Did not hear about model family	264(36.4)
Have heard but not at all working towards graduation	278(38.3)
Working towards graduation or graduated as model family	183(25.2)

### Utilization of maternal health services

More than half (67%) of the women had ever used contraceptives while 38% of them were current users. ANC visit at health facility was reported by 85% of the women. However less than half (48%) of the women had the World Health Organization (WHO) recommended 4 and more ANC visits. A small number (5%) of the women said that they gave birth at the health facility. A similar percentage (5 %) of the women had PNC check up. More than three quarters (85%) of the women had been tested for HIV. Iodized salt of greater than 15PPM was found in only 13% of the women’s households. Using the mean score for utilization of maternal health services as a cutoff point for good and poor utilization of maternal health services, about 37% of the women had good utilization of maternal health services while the rest had poor utilization.

### Who offers maternal health services?

The role of the HEWs to improved utilization of maternal health services is greatest in relation to family planning and ANC (Table 2). In regard to advice on family planning; 72% of the mothers reported to have received information on this topic from the HEWs. Forty-four percent of the mothers reported having been visited before delivery by HEWs. However, postnatal care and especially assistance during delivery still seem to be a big problem. The majority of the women (81%) delivered their baby with the help of relatives or friends and only 7% were assisted by the HEWs. Trained traditional birth attendants do better than HEWs in assisting births (20%).

**Table 2:** Women’s utilization of maternal health services by the type of health workers, September 2009 (N=725)

**Maternal health services**	**Frequency (no./%)**
**Family planning:** In the past 12 months, who among community health workers visited you and talked to you about family planning? *	
Health extension worker	523(72.1)
Community based reproductive health agent	88(12.1)
Community health agent	276(38.1)
Trained and untrained traditional birth attendant	331(45.7)
Not visited	145(20.0)
**Antenatal care:** Who (community health worker) visited you during your pregnancy of your youngest child?*	
Health extension worker	319(44.0)
Community health agent	98(26.1)
Community reproductive health agent	33(4.6)
Untrained or trained traditional birth attendant	157(41.8)
Was not visited or don´t remember	349(48.1)
**Delivery Service:** Who assisted you with the delivery of your youngest child?*	
Health professional	31(4.3)
Trained traditional birth attendant	147(20.3)
Untrained traditional birth attendant	29(4.0)
Relative/friend/neighbor	586(80.8)
Health extension worker	49(6.8)
No one	1(0.1)
**Post natal care:** If a community health worker visited you immediately after delivery of your youngest child, who was that person?*	
Health extension worker	189(26.1)
Community health agent	67(9.2)
Community based reproductive health agent	13(1.8)
Trained and untrained traditional birth attendant	170(23.4)
Was not visited or do not remember	389(53.7)

*Multiple responses were possible.

### Women’s utilization of primary care facilities for maternal health services

Health posts were rarely used by women for delivery services and PNC checkups (Table 3). Only 1% of the study participants gave birth at health posts. A similar percentage of participants had had PNC checkups for their baby at health posts. The utilization of health posts for family planning and ANC by women was relatively higher than for delivery. About 21% of the study participants had obtained contraceptives from the health posts. It seemed that women preferred the health center to the health post for ANC follow up (61% versus 23%).

**Table 3:** Women’s utilization of primary health care facilities for maternal health services, September 2009 (N=725)

**Maternal health services**	**Frequency (no./%)**
**Family planning:** Where did you obtain (current method) for the last time?	
Health center	117(16.1%)
Health post	150(20.7)
Others	7(0.8)
Non current users and pregnant mothers	451(62.2)
**Antenatal care:** Where did you receive antenatal care when you were pregnant for your youngest child?	
Health center	441(60.8)
Health post	163(22.5)
Others	9(1.3)
Did not go to a health facility	112 (15.4)
**Delivery service:** Where did you give birth for your youngest child?	
Home	691(95.3)
Hospital	10 (1.4)
Health center	16(2.2)
Health post	8(1.1)
**Post natal care**: After your youngest child was born, if a health worker checked your baby, where did that check take place?	
Your home	6(0.8)
Health post	10(1.4)
Health center	25(3.4)
Hospital	6(0.8)
Didn’t had check up	678(93.5)

### Comparison of findings of this study on utilization of maternal health services with findings of EDHS 2005

Compared to EDHS 2005 (Table 4), there is an increase in the proportion of women who have utilized family planning, antenatal care, and HIV testing. However, we observed no change in the proportion of women who have used health facility delivery and iodized salt.

**Table 4:** Comparison of findings of our study with finding of the 2005 EDHS for Tigray region and Ethiopia

**Access to maternal health services**	**National EDHS 2005 (%)**	**Regional Tigray EDHS 2005 (%)**	**Our study 2009 (%)**
Family planning current users	14.70	16.50	41.80
Antenatal care	27.60	35.30	84.60
Delivery at health facility	5.30	6.10	4.70
Postnatal check up	5.50	8.20	5.30
HIV ever tested	4.00	3.20	85.40
Iodized salt (>15PPM)	19.90	28.00	13.20

### Association of respondents’ characteristics with utilization of maternal health services

Calculated AOR through logistic regression analysis showed (Table 5) that women who were able to read and write (AOR, 1.85; CI 1.22-2.80), listened to a radio (AOR, 1.45; CI 1.05-2.02), had good participation in IGAs (AOR, 1.43; CI 1.03-2.00), and had been working towards graduation or graduated as model family (AOR, 2.13; CI 1.40-3.23) had good utilization of maternal health services. However variables including place of residence, age and year of enrolment didn’t show any significant association with good utilization of maternal health services.

**Table 5:** Association of respondents’ characteristics with utilization of maternal health services, September 2009

**Characteristics of respondents**	**Access to maternal health services**		**Adjusted Odds Ratio**	
	**Poor access to MHS**	**Good access to MHS**	**Odds ratio**	**95% CI-interval**
**District**				
Degua Tembien	165(68.8)	75(31.2)	1.00	
Saesi Tsadamba	157(64.3)	87(35.7)	1.19	0.81-1.74
Alaje	161(66.8)	80(33.2)	0.87	0.58-1.30
**Age of respondent**				
24 years and less	77(72.6)	29(27.4)	1.00	
25 and above	406(65.6)	213(34.4)	1.50	0.92-2.45
**Literacy**				
Illiterate : unable to read and write	397(68.9)	179(31.1)	1.00	
Literate : able to read and write	86(57.7)	63(42.3)	1.85*	1.22-2.80
**Listening to a radio**				
No, not at all	312(71.1)	127(28.9)	1.00	
Yes	171(59.8)	115(40.2)	1.45*	1.05-2.02
**Participation in income generating activities (IGAs)**				
Poor participation : 2 and less IGAs	324(71.1)	132(28.9)	1.00	
Good Participation : 3 and more IGAs	159(59.1)	110(40.9)	1.43*	1.03-2.00
**Year of enrolment into HEP**				
Do not know	117(73.6)	42(26.4)	1.00	
2004-2006	156(65.5)	82(34.5)	1.07	0.68-1.70
2007-2009	210(64.0)	118(36.0)	1.37	0.88-2.1
**Household status towards being a model family**				
Did not hear about model family	189(71.6)	75(28.4)	1.00	
Have heard about model family but not working towards graduation	202 (72.7)	76(27.3)	0.98	0.66-1.45
Yes, working towards graduation or graduated as model family	92(50.3)	91(49.7)	2.13*	1.40-3.23

*P<0.05.

## Discussion

Since the introduction of HEP in 2003 and deployment of HEWs, there has been an increase in the proportion of women who have utilized family planning, antenatal care, and HIV testing. On the other hand their deployment and work have not showed any improvement in utilization of health facilities for delivery, postnatal check up and use of iodized salt. Primary care facilities; particularly health posts, were almost unutilized by women for maternal health services. Women preferred to visit health centers instead of health posts. Women, who were literate, listened to the radio, participated in income generating activities and had been working towards graduation or graduated as model family were more likely to access and utilize comprehensive maternal health services.

Our finding on family planning is in agreement with other studies conducted in Ethiopia [11,12]. These studies showed HEWs have improved access to family planning. A study conducted in the southern part of Ethiopia found that women who were able to read and write are more likely to access maternal health services, similar to our findings. This study also showed similar to our findings on ANC that the proportion of women who had at least one ANC visit has increased considerably [13]. Nevertheless our study showed the proportion of women who had 4 and more ANC visits as recommended by WHO was still low (48%). Thus concerted effort by HEWs and VCHWs is necessary to educate women about the importance of having four and more ANC visits. Another important achievement observed in our study is the increase in HIV testing. A study on antiretroviral treatment in Ethiopia depicted a similar substantial expansion of access to HIV counselling and testing in Ethiopia [14]. This increase might not be totally attributed to HEWs, because nongovernmental organizations (NGOs) and other stakeholders also play a crucial role in HIV testing and education, through different approaches such as campaigns. HIV programs are highly supported by NGOs and other stakeholders. However, the positive role of HEWs in improving HIV testing and prevention in rural areas is undisputable. In reality, in rural kebeles in Ethiopia, HIV testing and education on HIV prevention is carried out primarily by HEWs. Even HEWs who are not trained for HIV testing organize and coordinate the campaigns for HIV testing. Practically all the health activities including campaigns at rural kebeles in Ethiopia are undertaken and organized by HEWs.

The HEWs did not succeed in improving utilization of health facility delivery, PNC check up and use of iodized salt. This calls for urgent interventions into the HEP. Innovative approaches are needed to improve HEWs effectiveness in relation to these services. Similar to our study, another study also showed no progress in skilled birth assistance and postnatal care coverage in Ethiopia since 1998 [12]. Contrary to the findings of a cross sectional study among 60 households in Tigray region which was conducted at the earlier stages of the HEP implementation, our study revealed the proportion of women who were assisted for birth by trained traditional birth attendants (TTBAs) is much higher than those assisted by HEWs [15]. This might be due to the fact that the number of TTBAs in a kebele is higher than the number of HEWs. It might be also TTBAs are tried and tested by women and seen to be experienced in conducting deliveries. Perhaps they could be closer and accessible to village women. On the other hand low competency and confidence of HEWs in assisting births, less favourable working conditions at the health posts, workload and walking long distances at night to assist births at home might also be attributed to this low performance of HEWs in assisting births [16].

Though further research is needed to study the HEWs’ performance in birth assistance, we propose several reasons for the present findings of their low participation in this role. First, health facility delivery is demanding in relation to cost, skill and competency. It requires HEWs having the necessary skills and communities having accessible and well-supplied facilities in place. Second, encouraging behavioural change for women to have births at health facility is time consuming work [17-19]. Women’s preference for having birth at home is a deeply embedded cultural belief. Women may believe that it is appropriate to go to a health facility for birth assistance and check up only if there are visible complications during birth [18]. Other determinants like women’s age, education, income, number of children and health seeking behaviour could also influence women’s preference on health facility delivery and birth assistance by skilled birth attendant [13]. Thus focused birth preparedness by pregnant women is necessary to encourage every woman to have birth at health facility or assisted by health professionals. It is advisable for HEWs and other community health workers to have effective discussion on birth preparedness with every pregnant woman when they do home based ANC visit. Third, health posts are not well equipped for providing delivery service which is a disincentive for women to use these facilities. Almost all health posts are a single room only, with no waiting room area, water source or electricity. Hence a strong referral system should be established between health posts and health centers (which are better equipped for birth deliveries) until health posts meet the necessary standards for delivery service. Fourth, HEWs’ low performance in assisting birth also relates to how HEWs are perceived by the community. The community may regard HEWs as less competent to assist birth. Unpublished reports from Tigray regional health bureau on the HEP indicate that the community perceive HEWs’ main task to be health education, sanitation and personal hygiene. Health extension workers were primarily associated with latrine construction. All these reasons and considering HEWs’ present workload and the poor conditions of health posts, it may be unrealistic to expect greater involvement in birth assistance by HEWs or that women would choose to give birth at health posts [16-20].

The 1978 Alma Ata Declaration on Primary Health Care [21], and subsequent reviews of primary health care reforms, call for intersectoral collaboration to address socio-economic determinants of community health, in which ensuring universal access to health services is one element [22,23]. In consideration of these other social determinants of women’s health, this study looked at whether participating in IGAs had an association with utilization of maternal health services. Logistic regression analysis revealed women who have been participating in three and more income generating activities were 1.72 times more likely to have good utilization of comprehensive maternal health services. The regression analysis also identified women who were literate, listened to the radio, and had been working towards graduation or graduated as model families for HEP were more likely to have good utilization of maternal health services. Hence these potential social factors could be targets for future intervention and support, as a means of increasing health care utilization. Year of enrolment into the HEP was not associated with good utilization of maternal health services. This may be due to the effect of diffusion of the intervention. Households who were enrolled later into the program may have opportunities to learn and share experience on positive health behaviours and information from households that were enrolled earlier.

### Strength and limitation of the study

Our study examined utilization of maternal health services among rural women who are difficult to reach. Our study is a cross sectional study and it may be difficult to attribute all the changes in utilization of maternal health services to the deployment of HEWs. Comparing our findings from a local sample with a national survey has its own limitation as the study population of our survey is small in size and from a specific region of the country while the national survey is large in size and representative for the whole country. Nevertheless, similar findings on improvements on utilization of family planning, antenatal care and HIV testing after the introduction of the HEP were observed by other studies conducted in other regions of the country. Thus the conclusions made in our study are most likely hold true not only for our study area but also for other areas in the country irrespective of the difference in socio-demographic characteristics across the country. It is worthy considering the way the outcome index (maternal health service utilization) is constructed, the variables chosen to construct this index and its categorization into good and poor. Had we chosen different variables and categorization to construct this outcome index, the results might have been different. Recall bias might also influence some of the results such as information on whether a household had been working towards graduation or graduated as model family or not and women’s involvement in IGA or not, because we took women’s word for these variables. Some important determinants of maternal health services utilization, for example distance to health facility, timing and frequency of HEWs visits, and household-decision making practices are not taken into account in our study. We recommend further study on the effect of these factors on utilization of maternal health services by rural women.

## Conclusions

This study has shown HEWs have brought essential maternal health care closer to the rural population in Ethiopia. Nevertheless their success is not for all components of maternal health services. HEWs brought improvement in utilization of family planning, ANC and HIV testing but not in assisting births. The perception that HEWs’ may be less competent in assisting births, the huge workload they already have, poorly equipped health posts and strong cultural beliefs supporting home births, make it unreasonable at the present time to expect substantial change in where and how women give birth. These challenging factors call for innovative strategies to support the efforts of HEWs in identifying risky mothers, birth preparedness and to improve their referral to health centers where midwives and better facilities for assisting births are available.

## Competing interests

The authors declare that they have no competing interests.

## Authors' contributions

AM and YK contributed to the design, data collection, analysis, and write up. NS, DS and YB contributed to the design, analysis and write up. MS, RB and DGJ contributed to analysis and write up. All authors read and approved the final manuscript.

## Pre-publication history

The pre-publication history for this paper can be accessed here:

http://www.biomedcentral.com/1472-6963/12/352/prepub
